# Knockdown of the type 1 cannabinoid receptor in the central amygdala increases both spontaneous and water deprivation-induced sodium intake in rats

**DOI:** 10.1152/ajpregu.00241.2024

**Published:** 2025-02-18

**Authors:** SG Ruginsk, MP Greenwood, M Greenwood, LLK Elias, D Murphy, J Antunes-Rodrigues

**Affiliations:** 1 Department of Physiological Sciences, Biomedical Sciences Institute, Federal University of Alfenas, Alfenas, Minas Gerais, Brazil; 2 Molecular Neuroendocrinology Research Group, Bristol Medical School: Translational Health Sciences, University of Bristol, Dorothy Hodgkin Building, Bristol, United Kingdom; 3 Department of Physiology, Faculty of Medicine of Ribeirao Preto, University of Sao Paulo, Ribeirao Preto, Sao Paulo, Brazil

**Keywords:** CB1 receptor, central amygdala, dehydration, natriorexigenic effect, extracellular fluid volume

## Abstract

Important inputs originating in the forebrain circumventricular organs and also in the central amygdala (CeA) trigger essential water deprivation (WD)-induced behaviors, such as thirst and sodium appetite. Together with the secretion of the neurohypophysial peptides vasopressin (AVP) and oxytocin (OT), these behavioral responses seek to maintain the normalcy of ECF osmolality and volume. Within this context, the main hypothesis tested by the present study was that CeA type 1 cannabinoid receptors (CB1Rs) were essential for the maintenance of body fluid homeostasis, particularly in response to WD challenge. We found that CeA CB1R knockdown increased spontaneous and WD-induced hypertonic saline intake, without significantly impacting water ingestion. In euhydrated rats, despite unaltered urinary volume, CB1R knockdown reduced urinary osmolality, as well diminished urinary nitrate concentrations, suggesting reduced renal sodium excretion. No relevant changes were induced by CeA CB1R knockdown on urinary parameters following WD-induced rehydration, which is consistent with unaltered AVP and OT mRNA transcription and hormone release under the same experimental conditions. Taken together, the present data support the notion that CeA CB1Rs participate in both spontaneous and WD-induced NaCl intake, without significantly affecting neuroendocrine output. Given the well described facilitatory CeA role on natriorexigenic responses, and the reported interplay between CB1Rs and gamma aminobutyric acid (GABA) within the CeA, the present findings suggest that CB1Rs may indirectly regulate sodium appetite through effects on CeA GABAergic neurotransmission.

## Graphical abstract

**Figure:**
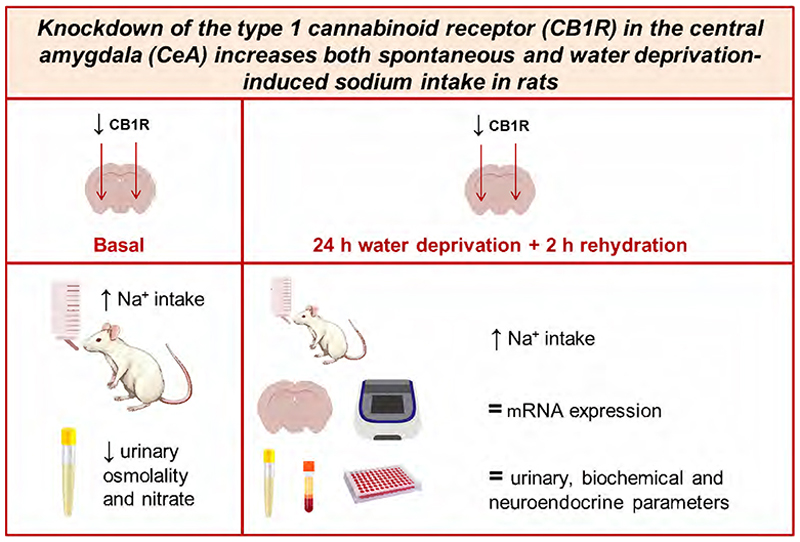

## Introduction

Body extracellular fluid (ECF) homeostasis is vital for maintenance of health, since any changes to ECF volume or composition may directly impact cellular function and survival. Therefore, when the organism is challenged with ECF disturbances, specific monitoring systems are activated in order to restore these vital parameters [1]. Centrally, the neuroendocrine reflexes regulating body ECF homeostasis are focused on the hypothalamic neurohypophysial system (HNS), which comprises large peptidergic magnocellular neurons (MCNs) of the supraoptic (SON) and paraventricular nuclei (PVN). The SON is a homogenous collection of MCNs, whereas the PVN is composed of distinct magnocellular groups, as well as smaller parvocellular neurons which project to the brainstem and spinal cord, regulating cardiovascular function [2].

A dehydration-induced increase in ECF osmolality activates the HNS either directly (through intrinsic MCNs osmosensitive properties) or indirectly (via specialized osmorreceptors located at the circumventricular organs, CVOs). MCN axons course though the internal zone of the median eminence and terminate on blood capillaries of the posterior pituitary gland, from where they release the two major neuroendocrine products, arginine vasopressin (AVP) and oxytocin (OT) into the systemic circulation. Dehydration also produces hypovolemia, which increases HNS sensitivity to hyperosmotic challenge, potentiating AVP release [3]. As part of the peripheral effector response, integrated AVP- and OT-mediated actions mediate a decrease in urinary output and the elimination of a highly concentrated urine, contributing to water conservation. In parallel, AVP also promotes vasoconstriction, in order to maintain blood pressure and adequate tissue perfusion [2].

The HNS not only receive important inputs from the brainstem and forebrain CVOs, as mentioned before, but also from other important brain nuclei, which trigger essential dehydration-induced behaviors, such as thirst and sodium appetite. It has already been demonstrated that the intracellular/extracellular dehydration chronologically activates either dipsogenic or natriorexigenic pathways, allowing the recovery of ECF tonicity, followed by the correction of ECF volume [4]. Within this context, the participation of the central nucleus of the amygdala (CeA) in the selective search for sodium has already been described in the literature. Accordingly, lesions of the CeA abolish ad libitum sodium intake in rats, without affecting elicited water consumption [5]. On the other hand, salt ingestion activates CeA neurons that express γ-aminobutyric acid (GABA), inhibiting further increments in NaCl intake [6]. Accordingly, intra-CeA administration of muscimol, a GABA-A receptor agonist, dose-dependently decreases NaCl ingestion induced by sodium depletion, an effect prevented by pre-treatment with bicuculline, a GABA-A receptor antagonist [7]. In parallel with the natriorexigenic response, treatment with GABA modulators also affects the secretion of natriuretic peptides, altering renal sodium handling [8].

More recently, the endogenously produced cannabinoids (eCBs) have been implicated in several neurotransmission events, directly modulating many homeostatic mechanisms, including ingestive behaviours and reward [9]. Evidence suggests that the type 1 cannabinoid receptor (CB1R) is the main isoform involved in the centrally-mediated eCB actions. Also, an important interplay between CB1Rs and GABAergic signaling has been already described - in the PVN, stimulation of CB1Rs determines an increase in local GABA release, culminating in an increased number of postsynaptic inhibitory potentials and, consequently, a decrease in neurosecretory function [10]. On the other hand, an opposite relationship between CB1Rs and GABA receptors has been proposed in the CeA. In a study performed by Varodayan and colleagues [11], the use of the CB1R agonist WIN decreased the frequency of postsynaptic currents mediated by the GABA-A receptor, an effect that was prevented by the CB1R antagonist.

Despite this evidence, the interplay between eCBs and GABA-mediated signaling within the CeA in the control of water/salt ingestion has not been yet investigated. Therefore, the main hypothesis of the present study was that CB1Rs could mediate CeA effects on salt intake. In order to test this hypothesis, we performed the intra-CeA post-transcriptional silencing of CB1R gene using a small hairpin interfering RNA (shRNA) carried by a viral vector. Experimental animals were evaluated under basal conditions and in response to a 24 h water deprivation (WD) challenge.

## Materials and Methods

### Animals

Adult male Wistar rats (*Rattus novergicus*) aged approximately eight weeks (250g) were obtained from Central Animal Facility from the University of Sao Paulo, Ribeirao Preto, State of Sao Paulo, Brazil. The animals were initially housed in collective cages (3-4 animals), with free access to tap water and standard rat chow, under controlled temperature (21 ± 2 ºC) and 12-h light-dark cycle (7 a.m./7 p.m.). After acclimatization for 2-3 d, the animals were submitted to the stereotaxic viral injections into the CeA. Postoperatively, the animals received a single prophylactic intramuscular dose of pentabiotic (Fort Dodge, 0.1 mL/100 g of body mass). All the procedures were conducted in accordance with the international guidelines for animal use in research (Guide for the Care and Use of Laboratory Animals, NIH Publication number 85–23, revised 1996), respecting the ethical principles of the Brazilian Society of Science in Laboratory Animals (SBCAL; available at <http://www.cobea.org.br>). This project underwent evaluation and approval by the Institutional Animal Research Ethics Committee of the Faculty of Medicine of Ribeirao Preto, University of Sao Paulo, Ribeirao Preto, State of Sao Paulo, Brazil.

### Lentivirus production, purification and titration

The *Rattus norvegicus* coding sequence for CB1R was obtained using the FASTA tool (http://www.ncbi.nlm.nih.gov). The primers were designed with the Block-itTM RNAi Designer tool and sequences encoding restriction sites for the Apa I and EcoR I enzymes were incorporated for subsequent insertion into the pSilencer 1.0-U6 vector. The position of the insert was confirmed by enzymatic digestion (Hind III). Then, the insert encoding the shRNA was amplified together with the U6 promoter region of pSilencer by a conventional polymerase chain reaction (PCR) and inserted into a lentiviral vector, in which the original PGK promoter region was replaced by CMV (pRRL.SIN.CPPT.CMV.IRES-GFP.WPRE). Both the lentiviral vector and the insert were digested using the enzymes Pac I and Xho I. After ligation, transformation was carried out into STBL3 bacteria (Invitrogen) and the construct was purified by Maxiprep (Invitrogen), obtaining a concentration of 2.25 μg/μL. In order to confirm the presence of the cassette of interest in the plasmid vector, double digestion was performed with the restriction enzymes PacI and XhoI (New England Biolabs) in CutSmart® buffer (New England Biolabs) at 37 °C for 16 h. For verification, the samples were subjected to electrophoresis in 1% w/v agarose gel, confirming the presence of two bands (7500 and 900 base pairs). A lentiviral vector expressing green fluorescent protein (GFP, pRRL.SIN.CPPT.CMV.GFP.WPRE) was used as a control (Addgene). Viruses were generated by transfection into HEK293T cells by the calcium phosphate method, as previously described [12]. Culture supernatant containing lentivirus was collected at 48 and 72 h after transfection, cell debris was removed by centrifugation, and the supernatant was filtered through a 0.45 μm filter (catalog #430770, Corning). High-titer lentiviruses were produced by centrifugation at 6000 × g for 16 h (400 mL), followed by ultracentrifugation of the resuspended pellet (10 mL of PBS) for 1.5 h at 50,000 × g. The viral pellet was resuspended in 150 μL of pre-warmed PBS and stored in 5 μL aliquots at – 80 °C. Viral titers were determined by counting GFP-positive cells at day 3 following infection of HEK293T cells.

### Central administration of the viral vectors

**Figure 1:**
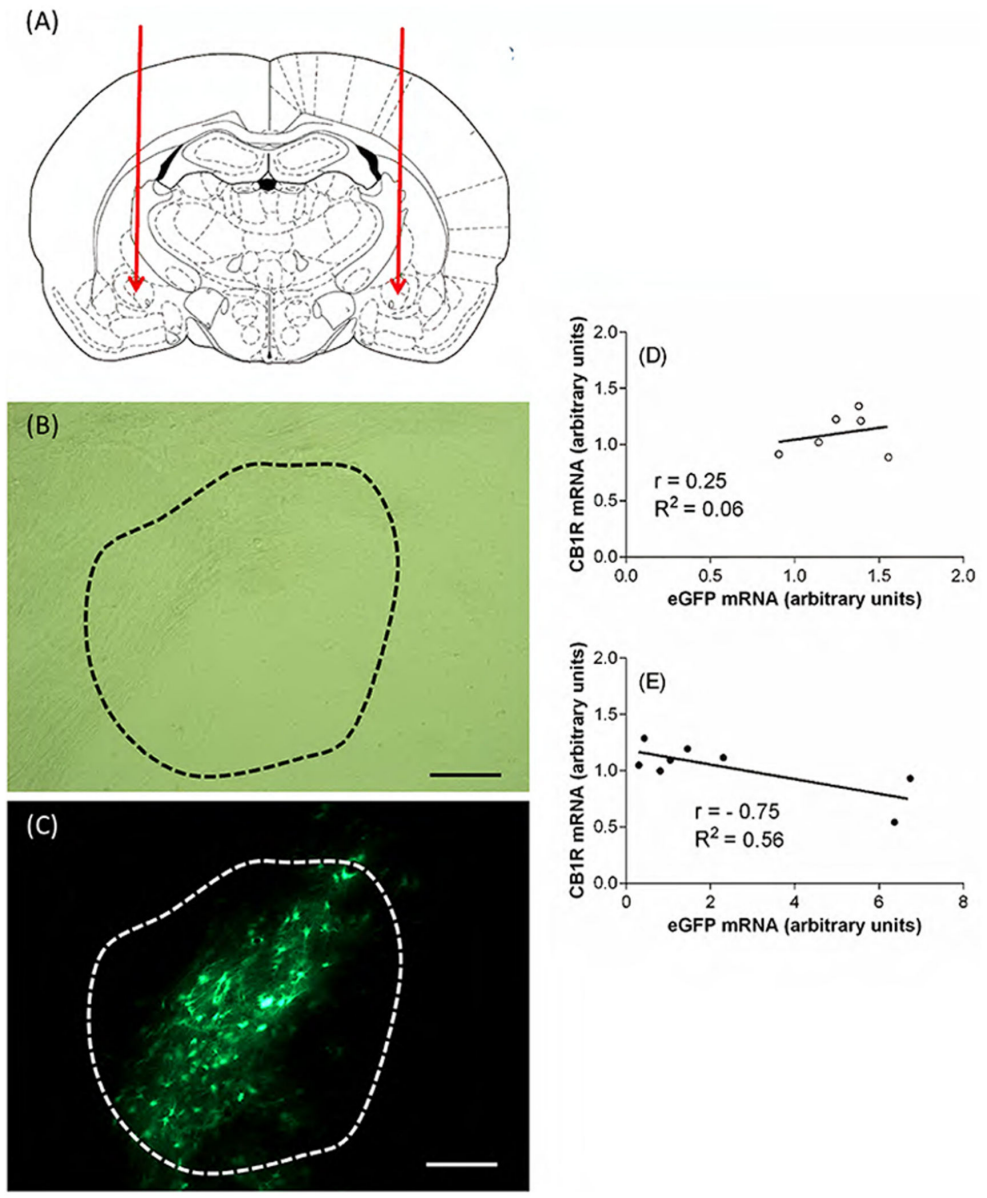
(A) Stereotaxic coordinates according to the rat brain atlas [13] for the intra-CeA administration of viral vectors carrying Scrambled or CB1R shRNA. The red arrows represent the bilateral injection sites; (B) Bright field photomicrograph of the CeA (unilateral); (C) enhanced green fluorescent protein (eGFP)-positive cells in the CeA, indicating viral infection; (D) eGFP mRNA expression (arbitrary units, X axis) was poorly correlated with type 1 cannabinoid receptor (CB1R) mRNA (arbitrary units, Y axis) in Scrambled animals (open circles), which is consistent with the unspecific transfection; (E) In CB1R knockdown animals (black circles), the greater the eGFP mRNA expression (arbitrary units, X axis), the lower CB1R mRNA expression (arbitrary units, Y axis), characterizing a strong inverse correlation. Data in Figures 1D and 1E were analyzed by linear regression (R^2^). The correlation coefficient has also been provided (Pearson, r). Scale bar: 100 µm.

The injections of viral vectors into the CeA were carried out in anesthetized experimental animals (xylazine 10 mg/kg and ketamine hydrochloride 90 mg/kg, 1 mL/Kg of body mass, intraperitonial). Animals were positioned in a stereotaxic apparatus and a 2 cm rostral–caudal incision was made to expose the skull surface. The anatomical coordinates for the CeA were adjusted (anteroposterior: 2.1 mm posterior to bregma; latero-lateral: 4.1 mm, bilateral; dorsoventral: 7.8 mm) and two 1 mm holes were drilled in the skull. An ultrafine pulled glass pipette was used to inject 1 µL of purified virus into each side of the CeA (Figure 1). Each injection was carried out over a period of approximately 20 min. Experimental animals received the injections of the CB1R shRNA, whereas control animals received the injections of a Scrambled shRNA. After 72 h of surgery recovery, animals were transferred to metabolic cages and were allowed to acclimatize for an additional period of 72 h. Fluid intake, food intake and urine output were recorded under basal conditions for the following 15 d. At the end of this period, animals were divided into 2 groups: euhydrated (free access to food and water) and 24 h WD (free access to food, but not to water). After the challenge, a cohort of Scrambled and CB1R shRNA WD animals had fluids (water and 0.3 M NaCl) reintroduced for 2 h. After decapitation or perfusion, the brains were removed and processed either for quantitative PCR (qPCR) or immunofluorescence, respectively. For qPCR experiments, the hit rates were confirmed by the evaluation of enhanced green fluorescent protein (eGFP) and CB1R mRNAs in micropunched samples of the CeA (Figures 1D and 1E). In immunofluorescence studies, the hit rates were evaluated by the anatomical confirmation of eGFP expression (green fluorescent signal, Figure 1C). Only animals which exhibited the simultaneous bilateral hits were considered for data collection.

### Determination of plasma hormones and hematocrit

For tissue collection, experimental animals were euthanized by decapitation. Trunk blood was collected into polypropylene tubes containing heparin (for determination of plasma AVP and OT concentrations) or into heparinized capillaries, which were centrifuged and read against a volumetric scale for hematocrit determination. Plasma OT and AVP concentrations were determined in previously extracted samples using in-house developed radioimmunoassays, as previously reported [14].

### Determination of urinary osmolality and nitrate concentrations

Urinary osmolality was measured on 100 μL of plasma or urine by freezing point depression using a micro-osmometer. For nitrate determinations, urine aliquots of 20 µL were deproteinized with 40 µL of absolute ethanol and kept at – 20 º C for 30 min. Next, they were centrifuged and the supernatant was applied to a chemiluminescent analyzer. In the present study, the assessment of urinary nitrate concentrations was used as an indirect estimative of renal nitric oxide (NO) production.

### Qualitative analysis of c-Fos expression by immunofluorescence

For this protocol, WD animals injected either with Scrambled or CB1R shRNA virus were perfused under deep anesthesia (xylazine 10 mg/kg and ketamine hydrochloride 90 mg/kg, 1 mL/Kg of body mass, intraperitonial) with heparinized PBS (pH 7.4, 150 mL) followed by paraformaldehyde (4% w/v PFA in PBS, 350 mL). The brains were removed, fixed for 4 h in the perfusion solution, and stored at 4 °C in PBS containing 30% w/v sucrose. Coronal sections of 30 μm were obtained in a cryostat and submitted to immunofluorescent labelling of c-Fos, using the free floating technique. Briefly, the sections were submitted to the blocking of nonspecific binding with normal donkey serum (10% v/v) in PBS for 1 h, followed by the overnight incubation at room temperature with the primary antibody anti-c-Fos (rabbit, 1:10,000, Oncogene Science). Thereafter, sections were rinsed and submitted to a 4-h incubation with the appropriate secondary fluorescent antibody (Cy5-conjugated donkey anti-rabbit, 1:250, Jackson ImmunoResearch). Sections were mounted on gelatinized slides using mounting medium and photographed under a Leica microscope equipped with a DC 200 Leica digital camera. Representative sections of the organum vasculosum of the lamina terminalis (OVLT) were acquired at the same level for 1 animal in each experimental condition (Scrambled or CB1R shRNA).

### Determination of mRNA expression by real-time quantitative PCR

After decapitation, brains were snap frozen on dry ice and stored at − 80°C. Thereafter, they were submitted to a 1-mm micropunch (Fine Scientific Tools) collection of the PVN. For this purpose, thick coronal sections were obtained in a cryostat and punched according to previously defined coordinates [13]. The samples were then dispensed into 1.5 mL tubes kept on dry ice. Total RNA was extracted from punch samples by combining Qiazol Reagent with Qiagen’s RNeasy kit protocols (Qiagen). The punch samples were removed from dry ice and rapidly resuspended, by vortexing, in 1 mL of Qiazol reagent. Following Qiazol phase separation with chloroform, 350 μL of the upper aqueous phase was removed, mixed with 350 μL of 70% v/v ethanol, and applied to RNeasy columns. The remaining steps were performed as recommended by the manufacturer. For cDNA synthesis, 500 ng of total RNA was reverse transcribed (High-Capacity cDNA Reverse Transcription, Applied Biosystems), diluted 1:5 in sterile water and assayed in triplicates (7500 Real-Time PCR System, Applied Biosystems) with the appropriate mixture of reagents (Master Mix, Applied Biosystems) and probes [β-actin: Rn00667869_m1 (VIC), OT: Rn00564446_g1 (FAM), AVP: Rn00566449_m1 (FAM), CB1: Rn00562880_m1 (FAM), eGFP: Mr04097229_mr (FAM), Applied Biosystems]. Data were obtained in real time and the relative expression of target genes in each sample was determined by either by the variation of threshold cycles (ΔΔCt) between target and housekeeping mRNAs or by the relative standard curve method, according to the assay efficiency. The results were expressed in arbitrary unites (relative to the control group).

### Statistical analysis

All data are expressed as the mean ± SEM. Statistical differences between experimental groups for basal parameters were evaluated using unpaired Student’s t test (parametric) or Mann-Whitney test (non-parametric). Two-way ANOVA followed by Sidak’s post hoc test were used to determine the differences between the groups submitted to WD and rehydration. p<0.05 (bicaudal) was considered significant.

## Results

### Effects of CeA CB1R knockdown on basal behavioral and renal parameters

**Figure 2:**
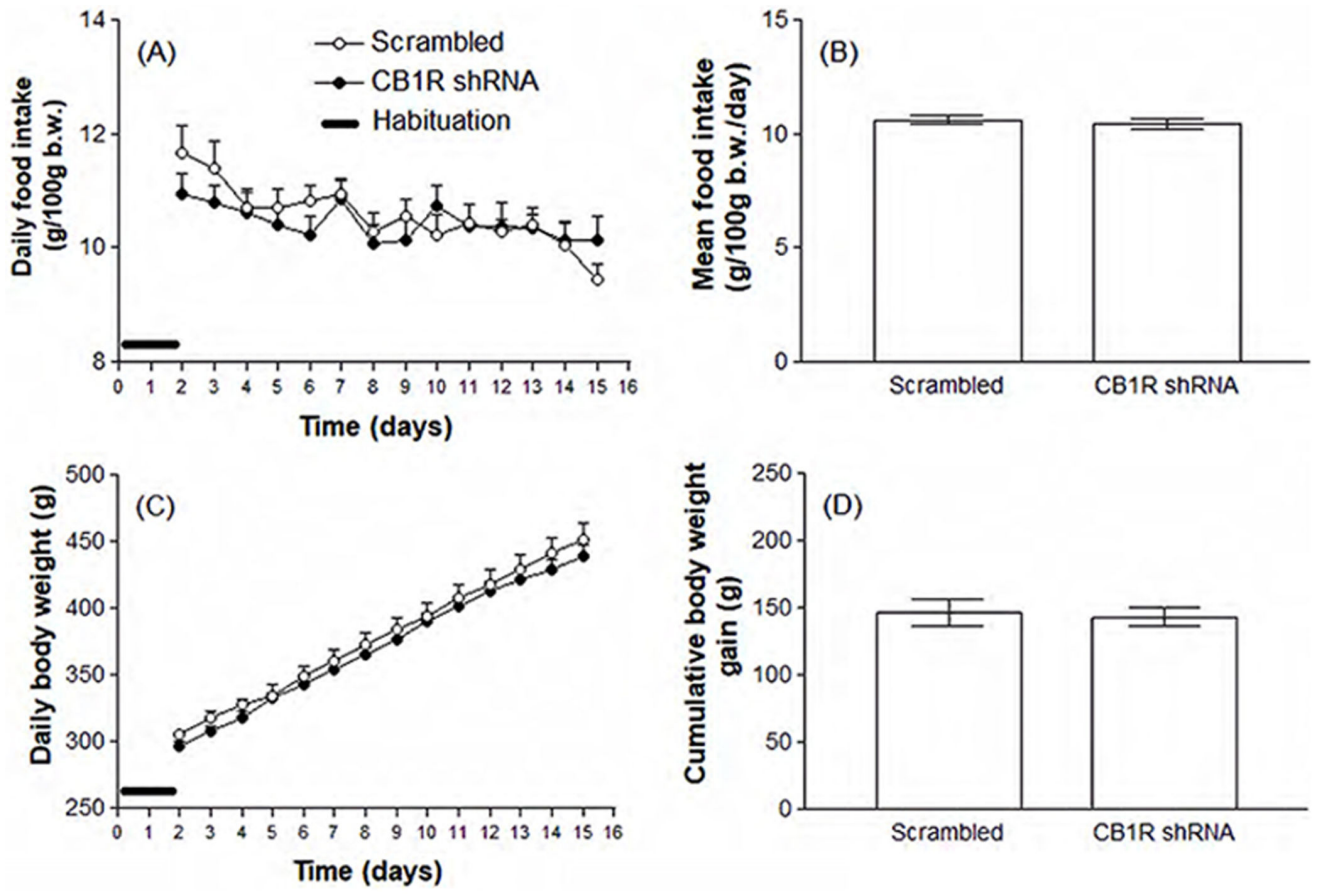
Daily spontaneous food intake (A and B) and body weigh variation (C and D) in animals previously submitted to the intra-CeA administration of viral vectors carrying Scrambled or CB1R shRNA. Results are expressed as mean ± SEM and were analyzed by unpaired Student’s t test. N = 8-11.

**Figure 3:**
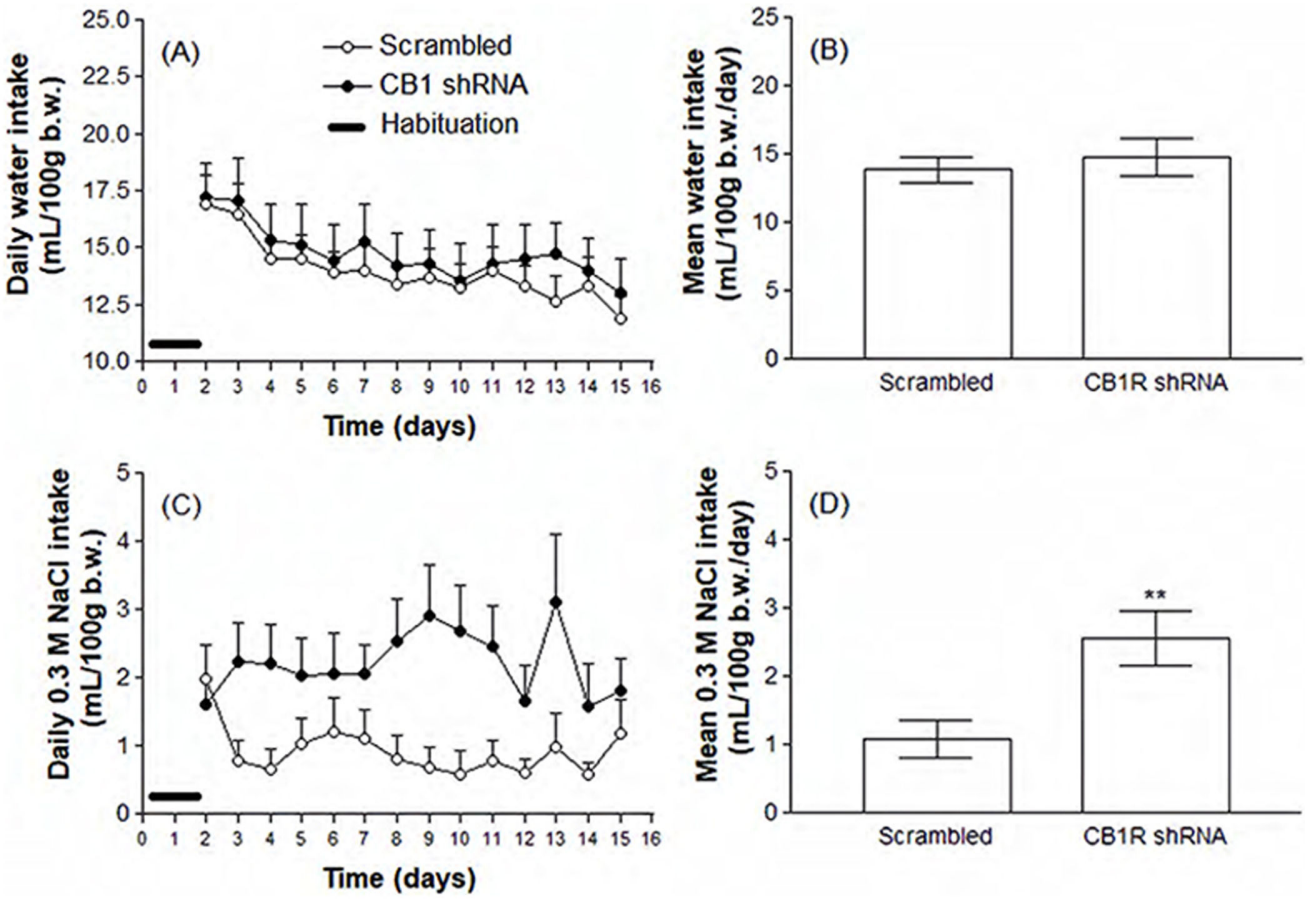
Daily spontaneous water (A and B) and 0.3 M NaCl intakes (C and D) in animals previously submitted to the intra-CeA administration of viral vectors carrying Scrambled or CB1R shRNA. Results are expressed as mean ± SEM and were analyzed by unpaired Student’s t test. ** p<0.01 versus Scrambled group. N = 8-11.

**Figure 4:**
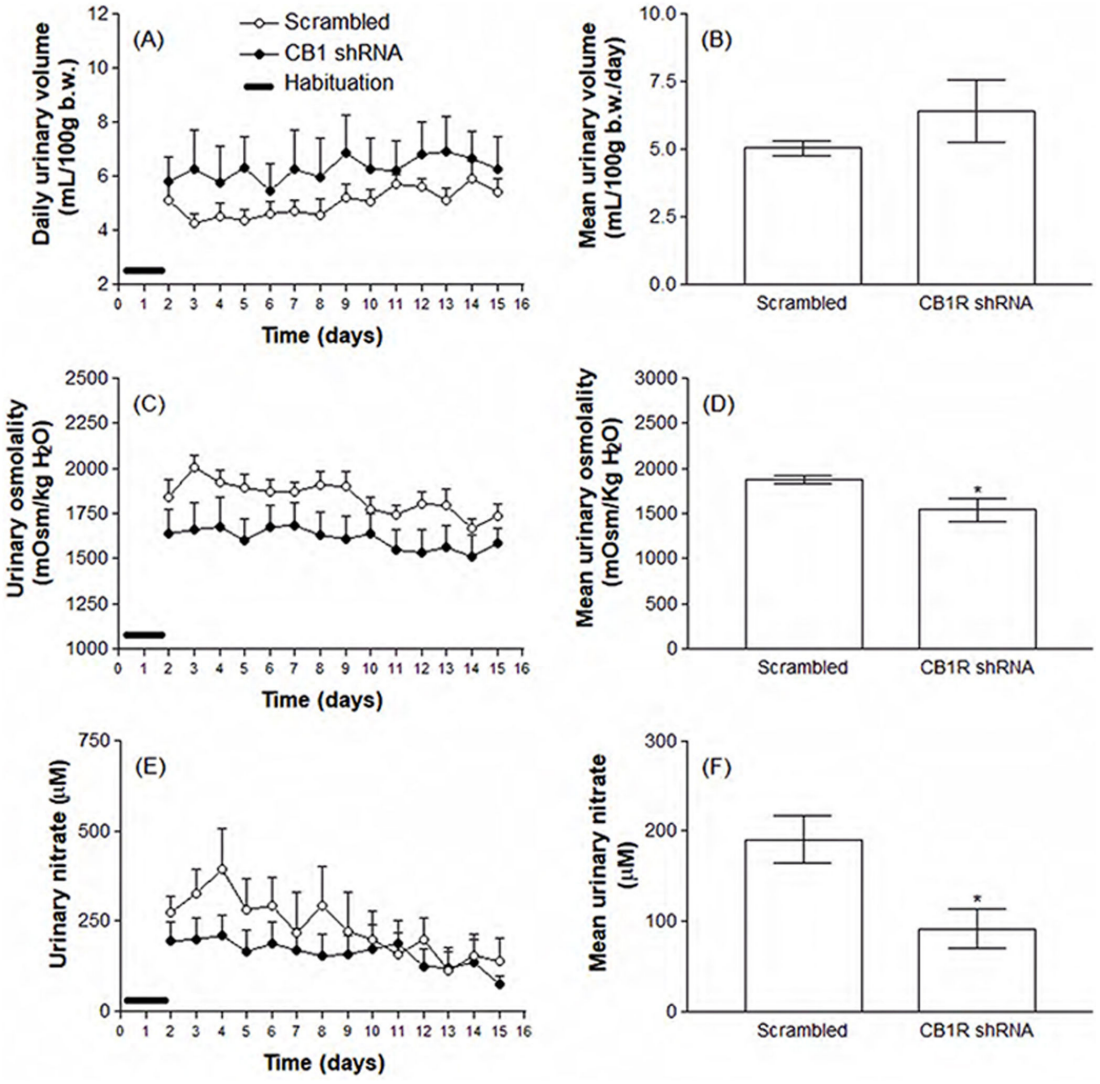
Daily spontaneous urinary volume (A and B), osmolality (C and D) and nitrate concentrations (E and F) in animals previously submitted to the intra-CeA administration of viral vectors carrying Scrambled or CB1R shRNA. Results are expressed as mean ± SEM and were analyzed by unpaired Student’s t test. ^*^ p<0.05 versus Scrambled group. N = 8-11.

Figure 2 shows that there was no significant change in body mass variation or food intake between animals injected into the CeA with the Scrambled shRNA (open circles), compared to animals that received the specific CB1R shRNA (black circles). Also, water intake was not significantly impacted by CB1R knockdown (Figures 3A and 3B). On the other hand, 0.3 M NaCl intake was significantly increased in animals injected with the CB1R shRNA (1.08 ± 0.27 versus 2.55 ± 0.39 mL/100 g b.w./day, p<0.01, Figures 3C and 3D). The effects of CB1R knockdown in the CeA on urinary parameters are shown in Figure 4. According to the results, animals knocked down for the CB1R exhibit a decreased urinary osmolality (1879 ± 45.71 versus 1544 ± 128.50 mOsm/Kg H2O, p<0.05) accompanied by decreased nitrate concentrations (190.6 ± 25.90 versus 91.71 ± 22.23 µM, p<0.05), although no significant changes were detected in daily urinary volume.

### Effects of CeA CB1R knockdown on behavioral, metabolic, neuroendocrine and renal parameters in WD rats

As shown in Table 1, Scrambled and CB1R shRNA groups showed similar food intake and similar lost weight when water-restricted. As expected, rehydration for 2 h induced significantly impacted hematocrit [F(1,10) = 23.18, p<0.001] and plasma osmolality [F(1,10) = 35.35, p<0.001]: hematocrit was restored by rehydration in both Scrambled and CB1R shRNA groups, whereas plasma osmolality was only significantly attenuated by rehydration in knockdown animals.

**Figure 5:**
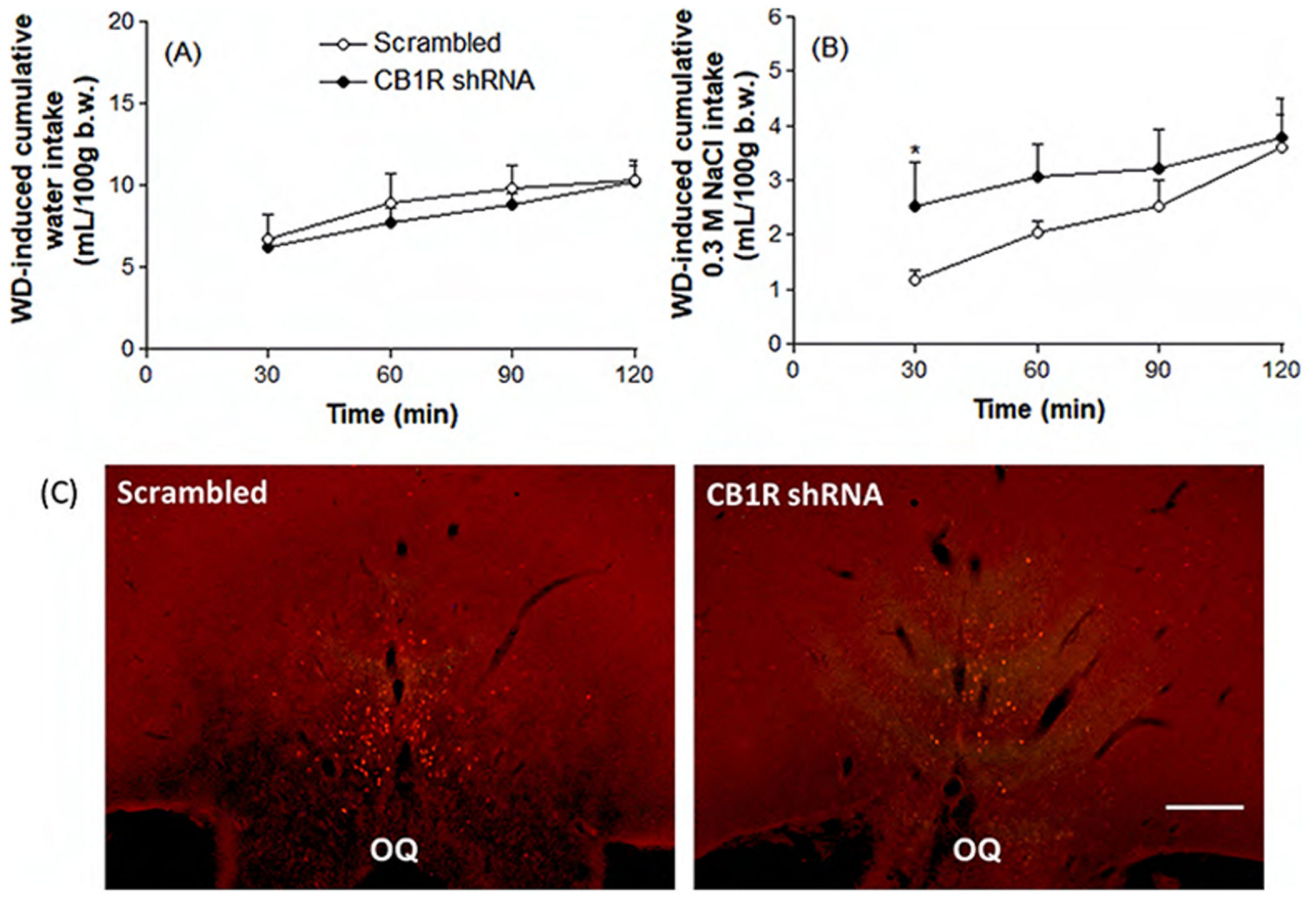
Rehydration-induced cumulative water (A) and 0.3 M NaCl (B) intakes in water deprived (WD) animals previously submitted to the intra-CeA administration of viral vectors carrying Scrambled or CB1R shRNA. In (C), a representative photomicrograph showing the qualitative evaluation of c-Fos immunoreactivity (in red) in the organum vasculosum of the lamina terminalis (OVLT) of both groups. Results are expressed as mean ± SEM and were analyzed by paired Student’s t test. ^*^ p<0.05 versus Scrambled group. N = 3-4. OQ: optic chiasm. Scale bar: 100 µm.

**Figure 6:**
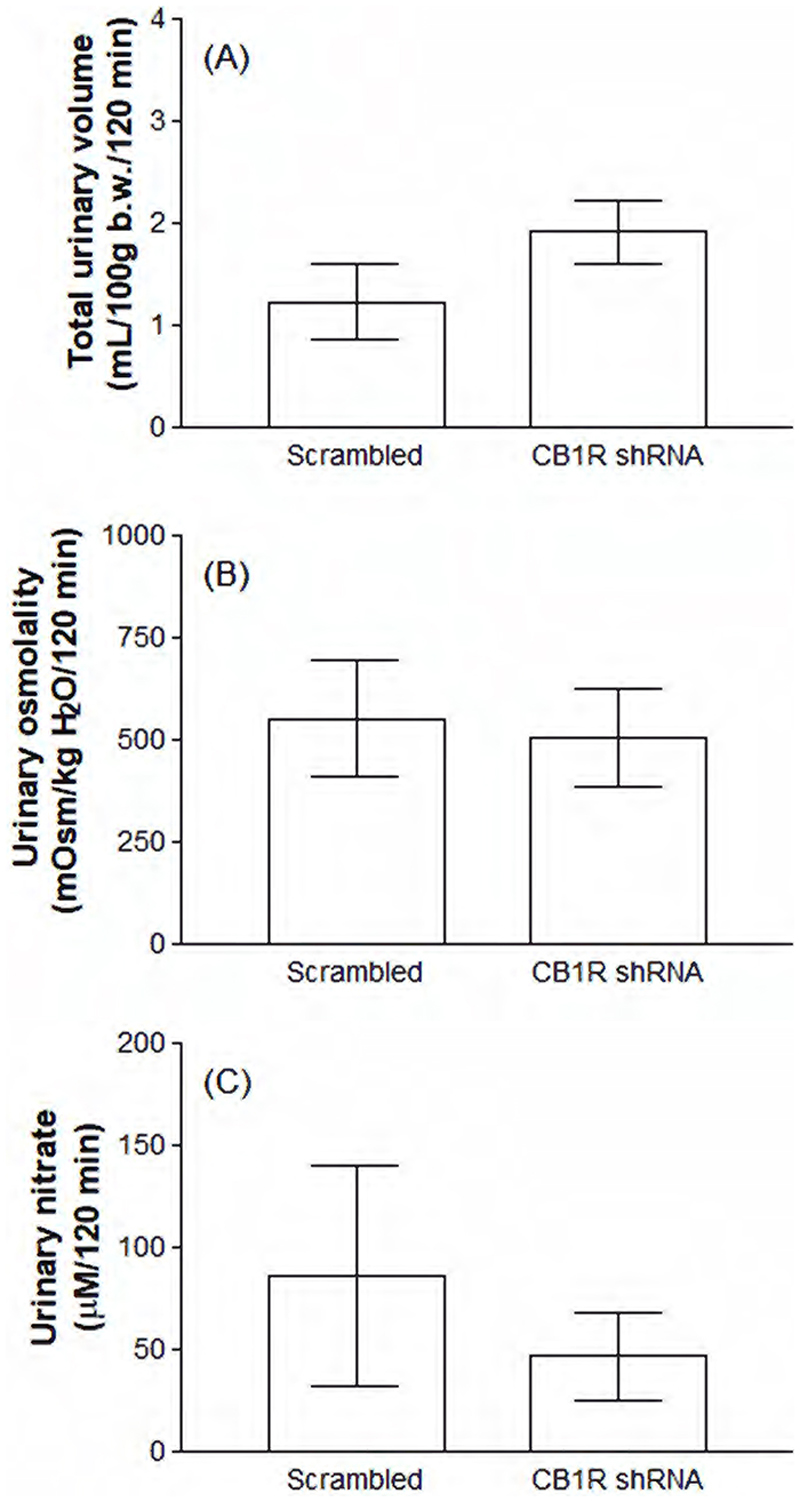
Rehydration-induced cumulative urinary volume (A), osmolality (B) and nitrate concentrations (C) in water deprived (WD) animals previously submitted to the intra-CeA administration of viral vectors carrying Scrambled or CB1R shRNA. Results are expressed as mean ± SEM and were analyzed by unpaired Student’s t test. N = 3-4.

Scrambled and CB1R knockdown animals challenged with WD were also evaluated for selective appetite for water (Figure 5A) and sodium (Figure 5B), as well as for urinary volume (Figure 6A), osmolality (Figure 6B) and nitrate concentrations (6C) after a 2-h period of rehydration with water and 0.3 M NaCl solution. The results show that there was no change between the groups in WD-induced water intake. However, animals that were subjected to CB1R knockdown initiated 0.3 M NaCl intake earlier than the control group (30 min: 1.18 ± 0.18 versus 2.52 ± 0.79 mL/100 g b.w., p<0.05), although the final volume of ingested saline did not differ between the groups. Interestingly, the number of c-Fos positive neurons in the OVLT of WD animals did not diverge between Scrambled and CB1R shRNA groups (Figure 5C). Finally, Figure 6 shows that, after rehydration, there were no significant changes associated with CB1R knockdown in urinary volume, osmolality or nitrate concentrations.

**Figure 7:**
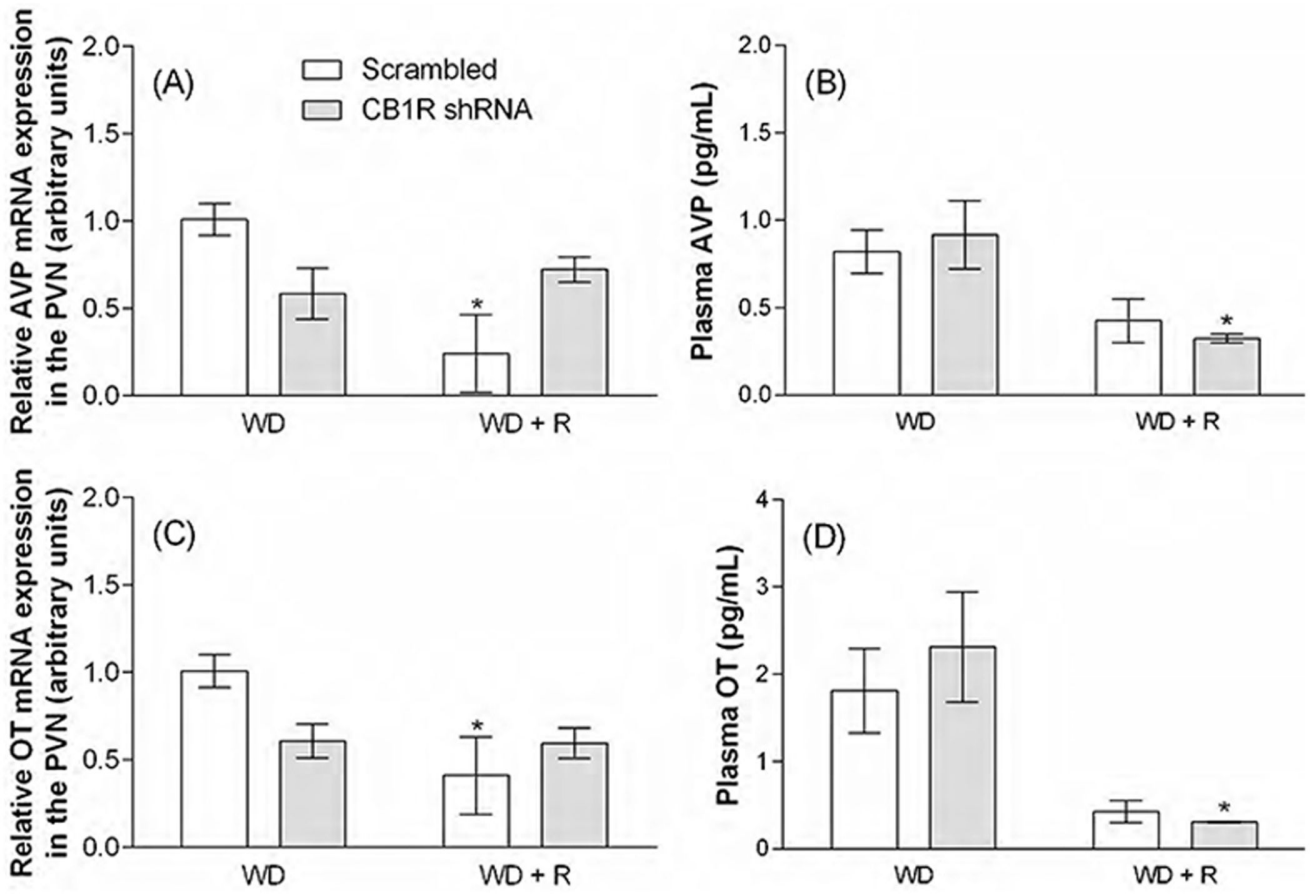
Rehydration(R)-induced vasopressin (AVP) and oxytocin (OT) relative mRNA expression (A and C) and plasma concentrations (B and D) in water deprived (WD) animals previously submitted to the intra-CeA administration of viral vectors carrying Scrambled or CB1R shRNA. Results are expressed as mean ± SEM and were analyzed by unpaired Student’s t test. ^*^ p<0.05 versus the respective Scrambled or CB1R shRNA WD group. N = 3-4. PVN: paraventricular nucleus of the hypothalamus.

Since AVP is the key hormone regulating water balance and OT is one of the main peptides controlling renal sodium excretion, we also investigated the effects of CeA CB1R knockdown on rehydration-induced mRNA expression and hormone secretion (Figure 7). According to the results, mRNA expression and neuropeptide release were similarly altered by WD in both Scrambled and CB1R shRNA groups. Rehydration with water and 0.3 M NaCl significantly impacted mRNA expression [AVP: F (1, 9) = 5.67, p<0.05; OT: F (1, 8) = 6.87, p<0.05], restoring transcripts to basal levels for both peptides in the Scrambled group [AVP: 1.00 ± 0.09 versus 0.24 ± 0.22 arbitrary units, p<0.05, Figure 7A; OT: 1.00 ± 0.09 versus 0.41 ± 0.22 arbitrary units, p<0.05, Figure 7C). Rehydration also resulted in restoration of AVP [F (1, 10) = 11.47, p<0.01] and OT [F (1, 8) = 16.93, p<0.01] plasma levels, although this effect was only statistically significant in the CB1R shRNA group (AVP: 0.91 ± 0.19 versus 0.32 ± 0.02 pg/mL, p<0.05, Figure 7B; OT: 2.31 ± 0.63 versus 0.30 ± 0.005 pg/mL, p<0.05, Figure 7D).

## Discussion

The present findings demonstrated for the first time that CeA CB1Rs are involved in the regulation of sodium balance. Interestingly, CeA CB1R knockdown increased spontaneous hypertonic NaCl intake, without significantly impacting water ingestion. Despite the unaltered urinary volume, CB1R shRNA animals showed reduced urinary osmolality, as well as increased urinary nitrate concentrations, suggestive of altered renal sodium management. CB1R knockdown also anticipated WD-induced natriorexigenic response. No relevant changes were induced by CeA CB1R knockdown on urinary parameters following WD-induced rehydration, consistent with unaltered AVP and OT mRNA transcription and hormone release under the same experimental conditions.

As outlined above, the hypothalamus, particularly the PVN, receives and processes a huge amount of information from numerous projections. These include visceral sensory information from the brainstem and from forebrain nuclei (such as the SFO), and also inputs from limbic regions, such as the amygdala and hippocampal formation. The amygdala comprises several smaller interconnected subnuclei which importantly participate in social, cognitive and affective responses [15]. The lateral amygdala receives information about the external environment from the sensory thalamus and cortices, and projects to the basal subnuclei (BLA), as well as to the neighboring central part (CeA). Therefore, whereas the BLA mostly convey sensory inputs, the CeA primarily mediates the conversion of these signals into output behaviors [16].

Within this context, amygdala CB1Rs are novel participants in water and sodium balance control. CB1Rs expressed by neurons located at the anterior cingulate cortex decrease glutamatergic drive onto BLA neurons, thereby promoting water intake [17]. More recently, it has been also demonstrated that the activity of BLA-projecting neurons originated in the posterior insular cortex is decreased in response to water drinking, whereas CB1R activation inhibits this circuitry, thus promoting a dipsogenic response [18]. Regarding sodium balance, several studies have already demonstrated a facilitatory natriorexigenic role for CeA neurons. However, when upstream intra-CeA GABAergic interneurons are activated, a decrease in sodium appetite is observed [5–7]. Other receptors (such as those mediating angiotensinergic, purinergic and opioidergic pathways) have also been shown to modulate CeA GABAergic output, thus modulating sodium appetite [19–21].

CeA CB1R knockdown consistently increased sodium, but not water intake in euhydrated rats. Although the intra-CeA GABA-mediated signaling has not been investigated, it can be speculated that the local decrease in CB1R-mediated signaling may produce a reduced GABAergic tonus [11], thus enabling sodium consumption. Also, in euhydrated CB1R knockdown animals, the spontaneous increase in 0.3 M NaCl intake was accompanied by a decrease in the total urinary concentration of osmolytes and nitrate, one of the main NO metabolites. Taken together, these data are consistent with evidence from the literature showing that decreased OT circulating levels may contribute not only to a decreased natriuretic effect at renal level, but also to a centrally-mediated decrease in salt appetite [22]. Accordingly, an increased NO renal production has been associated with the OT-mediated natriuresis [23], again supporting the present findings of parallel decreases in both urinary osmolality and urinary nitrate contents following hypertonic solution consumption.

We also demonstrated that the search for sodium is anticipated in CB1R knockdown animals subjected to WD. Indeed, osmosensory nuclei of the lamina terminalis project to the CeA, providing an excitatory drive for sodium intake, which is an essential behavioral response to guarantee ECF volume repletion [24, 25]. Since no important changes were observed in the WD-induced number of activated OVLT neurons between control and CB1R knockdown animals, we suggest that the facilitatory natriorexigenic response is probably generated by CeA downregulation of CB1Rs, rather than by an increased forebrain input.

When animals are challenged by WD, behavioral adjustments in water and sodium intakes occur. In parallel, important neuroendocrine outcomes targeting renal water conservation and increased sodium excretion take place, with the neuropeptide hormones AVP and OT being the major players within this context [3]. Although we have previously demonstrated that CB1Rs are essential for appropriate WD-induced AVP and OT secretion [26], no effects were produced by the CeA CB1R knockdown on neuropeptide production (mRNA) or release. We only observed a significant effect upon rehydration, irrespective of CB1R knockdown, in returning AVP and OT secretion to basal levels, as confirmed by the restoration of hematocrit and plasma osmolality in fluid replenished rats.

Finally, WD also triggers a clear hyperosmolality-induced anorexigenic effect, which is associated with parallel changes in the hypothalamic expression of peptidergic systems that regulate food intake [26]. Also, OT centrally suppresses food appetite, acting as a downstream mediator of other systems implicated in satiety-related responses [27, 28]. Together with the decreased ECF volume, the WD-induced reduced food intake contributes to the significant decrease in body mass observed in dehydrated rats (Table 1). Despite the suggestive interplay between CB1Rs and the WD-induced anorexia [26], no relevant changes were found in energy homeostasis parameters in response to CB1R knockdown.

Taken together, the present data support the notion that CeA CB1Rs participate in both spontaneous and WD-induced NaCl intake, without significantly affecting neuroendocrine output. Given the well described role of intra-CeA GABAergic neurons in inhibiting natriorexigenic responses, and the reported effect of retrograde CB1R-mediated eCB signaling in locally inhibiting GABA release, the present findings suggest that CeA CB1Rs may constitute an important system indirectly regulating sodium appetite, possibly through its effects on GABAergic neurotransmission.

## Figures and Tables

**Table 1:** Metabolic and fluid homeostasis parameters in 24 h water deprived (WD) Scrambled (control) and CB1R shRNA animals submitted to 2 h of rehydration (water + 0.3 M NaCl solution).

Group/Variable		Food intake (g/100 g b.w.)	Body mass variation (g/day)	Hematocrit (%)	Plasma osmolality (mOsm/Kg H_2_O)
24 h WD	Scrambled	6.54 ± 0.25	- 38.50 ± 3.72	54.25 ± 1.49	304.50 ± 3.12
	CB1RshRNA	5.49 ± 0.44	- 40.27 ± 3.41	57.25 ± 2.28	300.00 ± 2.41
24 h WD + 2 h rehydration	Scrambled	NA	NA	39.50 ± 6.50*	289.50 ± 3.50
	CB1RshRNA	NA	NA	39.72 ± 2.26**	283.50 ± 3.30*

Results are expressed as mean ± SEM and were analyzed by Two-way ANOVA, followed by Sidak’s post-test.

* p<0.05

** p<0.01 *versus* the respective 24 h WD group. N = 8-11 for food intake and body mass variation. N = 2-4 for hematocrit and plasma osmolality. CB1R = type 1 cannabinoid receptor; shRNA = small hairpin ribonucleic acid; WD = water deprivation.

## New & Noteworthy

CeA CB1R knockdown increased spontaneous and WD-induced hypertonic saline intake, without significantly impacting water ingestion. In euhydrated rats, despite unaltered urinary volume, CB1R knockdown reduced urinary osmolality, as well diminished urinary nitrate concentrations, suggesting reduced renal sodium excretion. No relevant changes were induced by CeA CB1R knockdown on urinary parameters following WD-induced rehydration, which is consistent with unaltered AVP and OT mRNA transcription and hormone release under the same experimental conditions.
